# Hierarchicell: an R-package for estimating power for tests of differential expression with single-cell data

**DOI:** 10.1186/s12864-021-07635-w

**Published:** 2021-05-01

**Authors:** Kip D. Zimmerman, Carl D. Langefeld

**Affiliations:** 1 Center for Precision Medicine, Wake Forest School of Medicine, Winston-Salem, NC USA; 2 Department of Biostatistics and Data Science, Wake Forest School of Medicine, Winston-Salem, NC USA; 3 Comprehensive Cancer Center, Wake Forest Baptist Medical Center, Winston-Salem, NC USA

**Keywords:** Hierarchical data, Single-cell, RNA-sequencing, Power calculator, Simulation, R-package, Mixed-effects models

## Abstract

**Background:**

Study design is a critical aspect of any experiment, and sample size calculations for statistical power that are consistent with that study design are central to robust and reproducible results. However, the existing power calculators for tests of differential expression in single-cell RNA-seq data focus on the total number of cells and not the number of independent experimental units, the true unit of interest for power. Thus, current methods grossly overestimate the power.

**Results:**

*Hierarchicell* is the first single-cell power calculator to explicitly simulate and account for the hierarchical correlation structure (i.e., within sample correlation) that exists in single-cell RNA-seq data. *Hierarchicell*, an R-package available on GitHub, estimates the within sample correlation structure from real data to simulate hierarchical single-cell RNA-seq data and estimate power for tests of differential expression. This multi-stage approach models gene dropout rates, intra-individual dispersion, inter-individual variation, variable or fixed number of cells per individual, and the correlation among cells within an individual. Without modeling the within sample correlation structure and without properly accounting for the correlation in downstream analysis, we demonstrate that estimates of power are falsely inflated. *Hierarchicell* can be used to estimate power for binary and continuous phenotypes based on user-specified number of independent experimental units (e.g., individuals) and cells within the experimental unit.

**Conclusions:**

*Hierarchicell* is a user-friendly R-package that provides accurate estimates of power for testing hypotheses of differential expression in single-cell RNA-seq data. This R-package represents an important addition to single-cell RNA analytic tools and will help researchers design experiments with appropriate and accurate power, increasing discovery and improving robustness and reproducibility.

**Supplementary Information:**

The online version contains supplementary material available at 10.1186/s12864-021-07635-w.

## Background

Robust and reproducible science depends on the quality of the experimental design. High quality experimental design revolves around focused research questions or hypotheses, appropriate and valid measures of the central variables related to these hypotheses, statistically sound analysis plans, and properly computed power analysis [1]. While power analyses for genetic association studies and bulk RNA-seq approaches are well-established [2–6], such analyses remain a challenge in single-cell RNA-seq studies due to intra-sample correlation inherent in these data [7]. Such within sample correlations exist because cells from the same individual share a common genetic and environmental background that often leads to greater similarity in gene expression among cells in the same sample. Therefore, gene expression measures among cells from the same sample have a hierarchical correlation structure where cells nested within an individual are not independent units. At present, the correlative (hierarchical) nature of these data is often neglected, in both power analyses and tests of hypotheses (e.g., differential expression) [7]. This was recently highlighted in a valuable paper by Andrews et al. which states that “current single-cell differential expression tests treat each individual cell as a biological replicate and cannot account for shared genetic backgrounds or disease state”. [8] Ignoring the hierarchal nature of single-cell RNA-seq data leads to studies that are under powered and inappropriately analyzed [7], leading to incorrect inference, poor reproducibility, and financial investments in those errors. A contributor to these flawed practices is the void of single-cell specific methods and literature that properly account for this hierarchical structure. However, just as Andrews et al. pointed out, we – too - expect that, “as scRNA-seq is applied to larger cohorts and comparison studies, [there will be] further developments that lead to more accurate statistical models for more complex experimental designs.” An excellent starting place for more accurate statistical models are accurate power calculations for improved study design.

Besides the classic, closed form, normal theory power calculations (e.g., ANOVA) that make too many overly simplistic assumptions (e.g., normality, independence), the power calculators for testing single-cell RNA differential expression all simulate cells independently, without the within-subject correlation structure [9–11]. Previously, we documented that in tests of differential expression in single-cell RNA-seq data one needs to account for the within experimental unit (e.g., individual) and showed that mixed-effects models with subject/individual as a random effect is a practical and statistically sound approach for these hypotheses [7]. Here, we present an R-package, *Hierarchicell*, with two purposes: 1) it is a simulator of hierarchical single-cell RNA-seq data, and 2) it computes power estimates using a mixed-effects models for testing hypotheses of differential gene expression in single-cell RNA-seq data. *Hierarchicell* simulates single-cell RNA-seq data with a hierarchical structure that closely resembles that of real data and can be used by researchers to make informed choices on experimental design while balancing the trade-off between cost and power. Our R-package is user friendly and flexible to a variety of scenarios. It incorporates estimates from real data [12] or allows users to input data (e.g., either Fluidigm C1 or 10x Chromium technology, user’s own pilot data) to obtain highly translatable and accurate estimates of power tailored to their technology. Within a well-characterized set of parameters that are modeled from either a user-defined or the default single-cell RNA-seq data, the tool provides users with estimates of power relative to a given fold-change, significance threshold, number of independent samples, and number of cells per independent sample. In addition, the calculator allows for the simulation of either continuous or binary phenotypes of interest. For binary case-control analyses, the user specifies the fold-change they desire to detect. For continuous phenotypes, the user specifies the mean and standard deviation of the phenotype and the degree of correlation with expression the user desires to detect with significance. Currently, most single-cell power calculators only provide estimates for the required number of cells rather than the required number of independent experimental units (e.g. individuals) or are not designed for computing power to detect differences in expression [13–15]. Other power calculations for single-cell RNA-seq are based on bulk RNA-seq methods to estimate the required number of samples [2, 3]. Estimating power for a single-cell RNA-seq study using bulk RNA-seq power calculators is a reasonable solution, but will underestimate the study’s power by not incorporating the additional power gained by sequencing numerous cells per individual. This tool provides a valuable resource in an area of critical need for researchers looking to optimize their study’s power and experimental design relative to the hierarchical nature that exists in all single-cell data.

## Implementation

### Overview of the *Hierarchicell* simulation engine

A step-by-step overview of the simulation procedure is provided with R-code examples and detailed explanations in *Hierarchicell*’s accompanying vignette. We encourage users to review this vignette (available on GitHub and in the Supplementary Materials) before beginning to work with *Hierarchicell*. The single-cell data in that example are used to estimate default simulation parameters for our simulation engine. These data were downloaded from the public accession number E-MTAB-5061 [12]. These data were sequenced using the Smart-Seq2 protocol and they include sequence data from 3514 cells from 10 different individuals [12]. Genes were previously normalized to account for the differences in library size [12]. After filtering down to high quality alpha cells, our dataset contained gene expression values for 22,983 genes and 886 cells (across 10 individuals). This dataset is included as part of the R-package for a number of reasons. Primarily, these data demonstrate the general intra- and inter-individual correlation patterns seen across a variety of single-cell data of different cell types generated by different platforms [7]. In addition, these data are not too large, allowing for the rapid estimation of simulation parameters while also minimizing the size of the R-package.

**Fig. 1:**
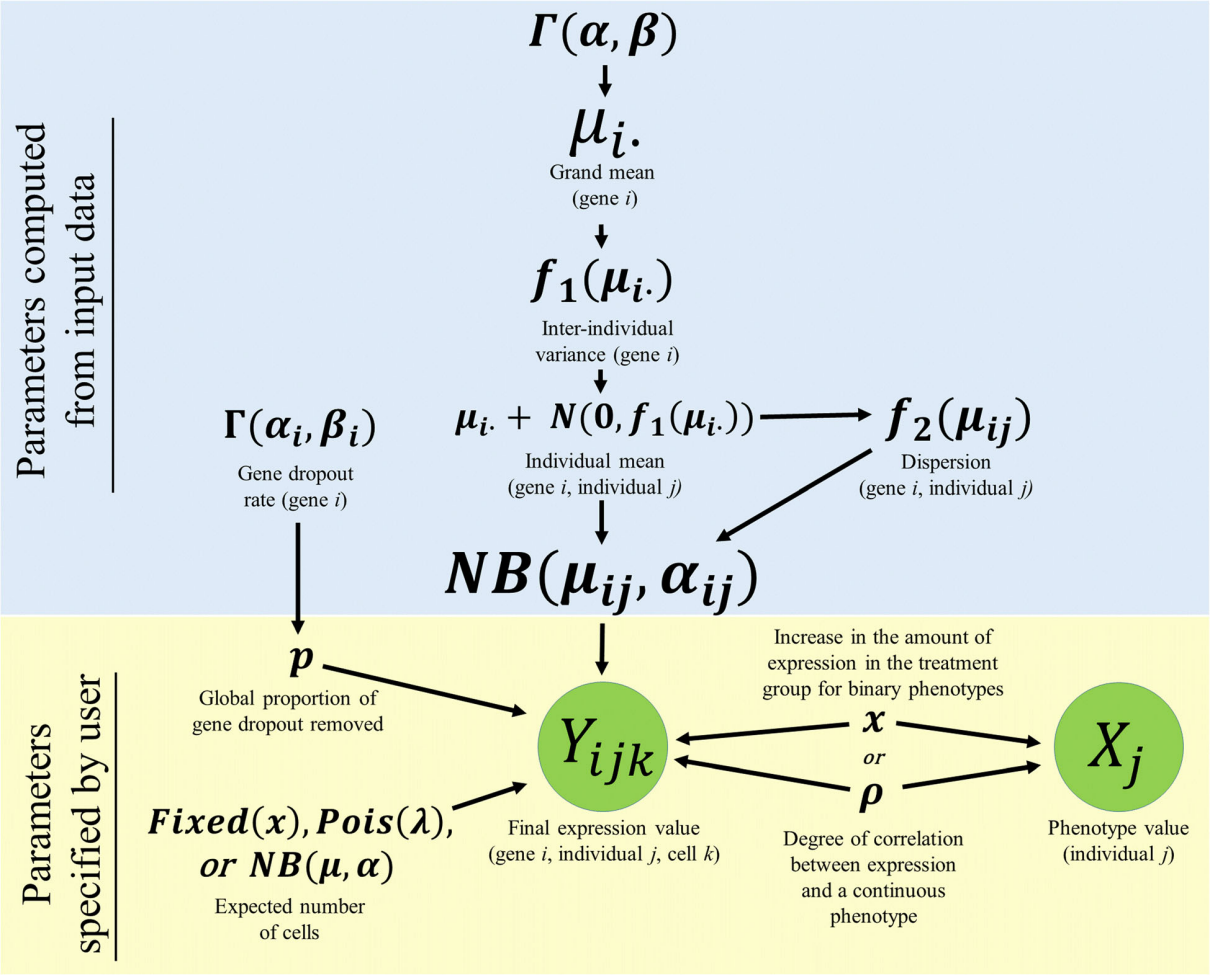
Overview of the hierarchicell simulation engine. The simulation procedure begins by estimating parameters from input data (blue) and then combines that information with parameters specified by the user (yellow) to simulate an expression value, *Y*_*ijk*_, for each gene *i*, individual *j*, and cell *k*

The simulation procedure was designed to simulate independent genes in a way that approximates the hierarchical structure of real data by empirically estimating the range of parameters (i.e., grand mean of the transcript-per-million (TPM) values, within sample variance, between sample variance, relationship between the grand mean and dispersion, dropout) that define the observed distribution of TPM values for a gene. To estimate these parameters, genes were pruned to a set of uncorrelated genes that captured the most representative patterns of detectable TPM values, without the resulting parameter estimates being primarily driven by dropout. Specifically, genes were sequentially sampled one at a time and any other gene having transcript abundances correlated (Spearman’s correlation coefficient > 0.25) with the gene were removed. To estimate the grand means independently from the hierarchical correlation structure, the grand means were estimated by sampling one cell from each individual and computing the mean TPM value 1000 times. The mean of each of those means was used to approximate the grand mean. To approximate the variance of the within-sample means (inter-individual variance), the means of all non-zero TPM values were computed across all cells within each individual and the variance between those values was subsequently computed. To estimate the within-sample dispersion values, the non-zero TPM values were first used to compute a within-sample variance and within-sample mean. Consistent with the classical definition of the Negative Binomial distribution’s dispersion parameter, the within-sample dispersion parameter was then computed as:

1 $$ {\alpha}_{ij}=\frac{{\mu_{ij}}^2}{\sigma_{ij}^2}-{\mu}_{ij} $$

where *α*_*ij*_ represents the dispersion parameter, *μ*_*ij*_ represents the within-sample mean, and ${\sigma}_{ij}^2$ represents the within-sample variance for gene *i* and individual *j*. The grand means and variances were computed empirically from the TPM values previously reported in six different cell types across three different single-cell studies [12, 16, 17]. Once consistent patterns were identified across cell types, alpha cells from the pancreas dataset, were used as the model data for our simulation. A gamma distribution was fit to the global mean of the TPM values of each gene using maximum-likelihood estimation. For each independently simulated gene *i*, a random value was sampled from this gamma distribution to obtain a grand mean, *μ*_*i*_. The variance of the within-sample means (inter-individual variance) was modeled as a linear function of the grand means, *f*_1_(*μ*_*i*_) and the within-sample dispersion (intra-individual variance) was estimated as a logarithmic function of the within-sample means, *f*_2_(*μ*_*i*_), and the probability of dropout was estimated independently as a bounded gamma distribution (Fig. 1). Using a normal distribution with an expected value of zero and a variance computed by the first linear relationship, *f*_1_(*μ*_*i*_), a difference in means was drawn for each of the individuals *j* in the simulation. This difference was summed with the grand mean to obtain an individual mean, *μ*_*ij*_. Three different methods were used to simulate the number of cells per individual. To simulate scenarios where each of the individuals had the exact same number of cells, the number of cells desired for each individual was fixed at a constant value. In order to simulate scenarios where the number of cells per individual demonstrated slight imbalance, a Poisson distribution with a λ equal to the expected number of cells desired for each individual was then used to obtain the count of cells for each individual. To simulate scenarios where the number of cells per individual demonstrated greater imbalance, the number of cells per individual were modeled as a Negative Binomial random variable with a mean equal to the expected number of cells and a dispersion parameter of one. For each gene *i* and cell *k* assigned to an individual *j*, a read count value, *Y*_*ijk*_, was drawn from a Negative Binomial distribution with an expected value equal to the individual’s assigned read count value, *μ*_*ij*_, and a dispersion parameter, *α*_*ij*_, computed by the logarithmic function of the grand mean *f*_2_(*μ*_*i*_).

### Overview of *Hierarchicell* power calculations

To compute power, transcripts-per-million (TPM) values were simulated for each gene with the user-specified fold-change or ρ parameter (Fig. 1). Fold-change should be specified where users are interested in computing power for two distinct groups. The ρ parameter, which represents the degree of correlation between gene expression and a simulated continuous phenotype, should be specified where users are interested in computing power for association analysis with a continuous trait. Here, fold-change is a constant that was multiplied by the global mean gene expression values to spike the expression of those genes in the desired treatment group. The direction of the fold-change was simulated with a Bernoulli distribution with a probability of 0.5 to allow the direction of effect to vary equally between groups.

We applied a two-part hurdle model with a random effect for individual as directed in MAST’s reference manual (7,18). Specifically, a log(x + 1) transformation of the data was applied and the hurdle model computed to find genes exhibiting differences in expression. Using their same notation, the addition of random effects for differences among persons is as follows:

$$ logit\left(\Pr \left({Z}_{ig}=1|{X}_i\right)\right)={X}_i{\beta}_g+{W}_i{\gamma}_j $$

2 $$ \Pr \left({Y}_{ig}=y\right|{Z}_{ig}=1\Big)=N\left({X}_i{\beta}_g+{W}_i{\gamma}_j,{\sigma}_g^2\right) $$

where *Y*_*ig*_ is the expression level for gene *g* and cell *i*, *Z*_*ig*_ is an indicator for whether gene *g* is expressed in cell *i*, *X*_*i*_ contains the predictor variables for each cell *i*, and *W*_*i*_ is the design matrix for the random effects of each cell *i* belonging to each individual *j* (i.e., the random complement to the fixed *X*_*i*_). β_*g*_ represents the vector of fixed-effects regression coefficients and γ_*j*_ represents the vector of random effects (i.e., the random complement to the fixed β_*g*_). γ_*j*_ is distributed normally with a mean of zero and variance ${\sigma}_g^2$. To obtain a single result for each gene, likelihood ratio or Wald test results from each of the two components are summed and the corresponding degrees of freedom for each component are added. These tests have asymptotic χ^2^ null distributions; these statistics can be summed and remain asymptotically χ^2^ because *Z*_*g*_ and *Y*_*g*_ are defined conditionally independent for each gene. When summed together, these tests provide a single test for the two-part hurdle model. Our package also offers the ability to compute type 1 error rates (and thereby power) for a variety of different single-cell analysis approaches. New methods that properly handle within sample correlation will be integrated as they become available.

### Software implementation

All simulations and data were compiled in RStudio using R-3.6.2 and is freely available on GitHub. The supplementing dataset that is included to run the R-package without user input data was significantly downsized by removing all of the genes correlated with a Spearman’s correlation coefficient > 0.25. This filtering is one of the first steps in our simulation procedure and doing so greatly reduced the size of the source package to 475 KB as well as the data structures held in memory during use. Currently, the simulation typically completes in less than 5 seconds, depending on user specifications. The simulation-based power calculations, however, can take much longer depending on the model that is used. For the recommended two-part hurdle mixed model (MAST with a random effect for individual), this can range anywhere from 1 to 20 min per simulation-based estimate of power for a given fold-change on a 64-bit Operating system with 8 CPUs and 16 GB of RAM. We note that these run times are heavily dependent on the number of genes, the sample size, the number of cells per individual specified, and the number of CPUs available.

## Results and discussion

Previously, we demonstrated that our simulation recapitulates the most important aspects of single-cell gene expression data, particularly the hierarchical structure of single-cell RNA-seq data which is rarely accounted for in differential expression analysis [7]. We also applied our simulation engine to demonstrate that mixed models are a statistically sound method that accounts for the within sample correlation and has appropriate type 1 error control without sacrificing power [7]. In addition, we provided power estimates for binary outcomes across a range of experimental conditions to assist researchers in designing appropriately powered studies [7].

**Fig. 2:**
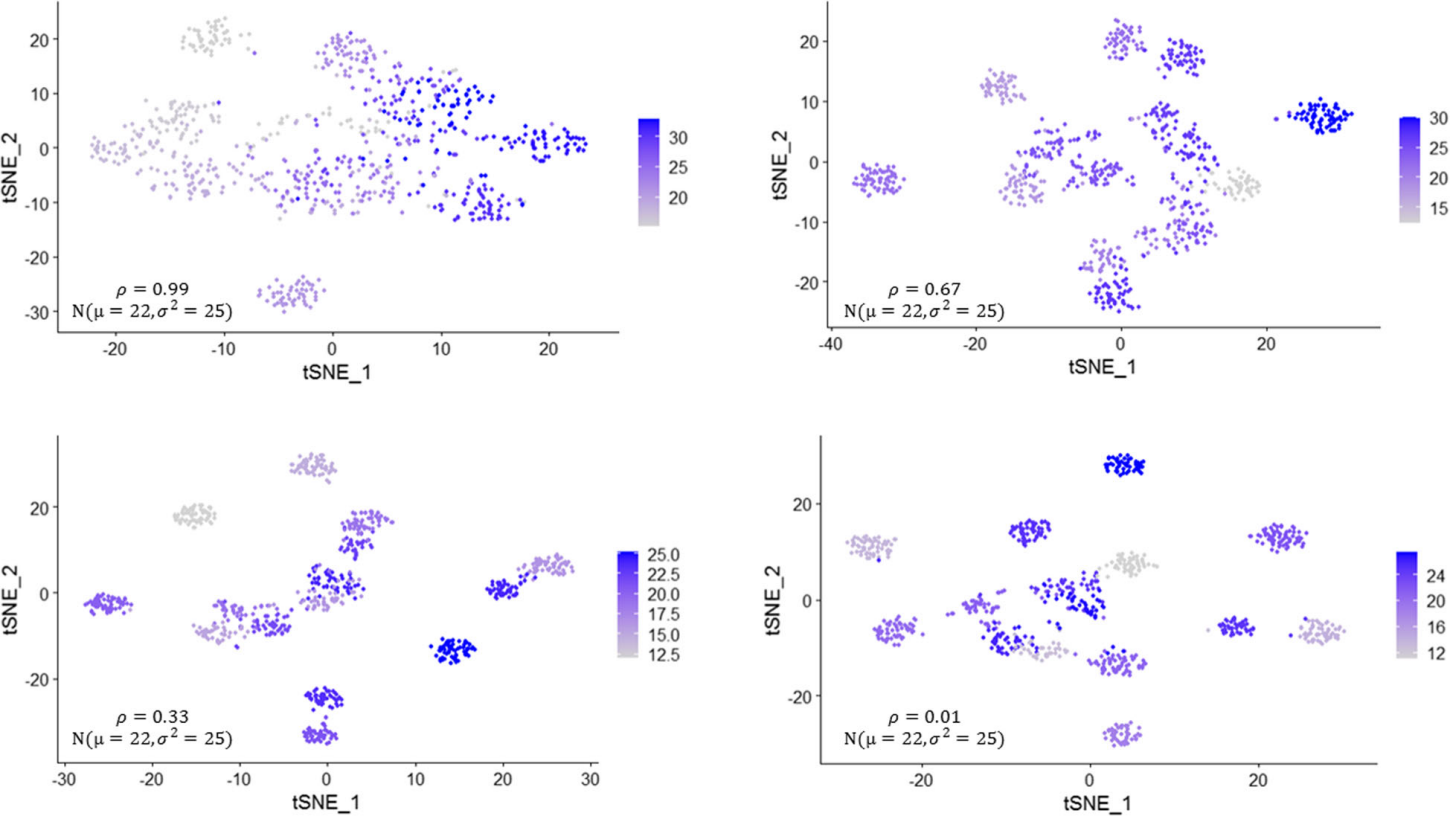
tSNE plots of gene expression data simulated to correlate with a continuous variable. The continuous phenotype is simulated with normal with a mean of 22 and standard deviation of 5 and correlates at various levels with gene expression. In the top left panel, the correlation between gene expression and the simulated phenotype is 0.99. In the top right it is 0.67, on the bottom left it is 0.33, and on the bottom right it is 0.01

We previously reported power calculations for tests of differential expression in single-cell RNA-seq studies for binary phenotypes (i.e., case/control treatment groups) [7]. *Hierarchicell* now also allows users to estimate power for detecting associations between continuous traits and single-cell gene expression (Fig. 2). The simulated expression data can be computed over a range of correlations with the magnitude of expression in each individual’s cells, while accounting for the hierarchical structure of these data.

**Fig. 3:**
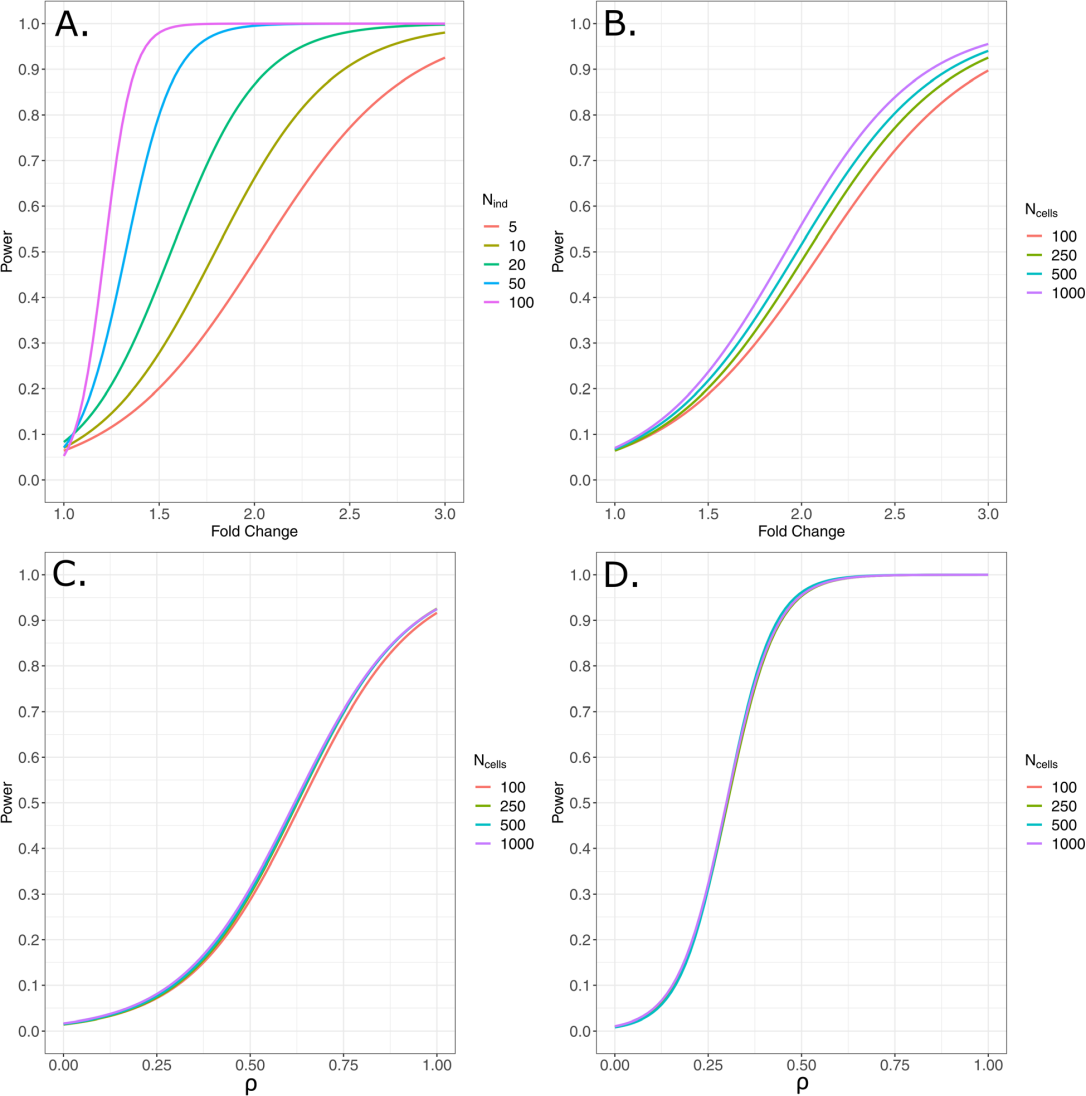
Power calculations using MAST with a random effect for individual. Power curves for various, but likely, single-cell scenarios using MAST with a random effect for individual. Power is computed at α = 0.05. Panel **a** demonstrates differences in power when sample sizes range between 5 individuals per group to 100 when the number of cells per individual is held constant at 250. Panel **b** demonstrates the differences in power when increasing the number of cells per individual (100, 250, 500, 1000) for 10 individuals per group. Panels **c** and **d** demonstrate the very minor differences in power by increasing the number of cells per individual (100, 250, 500, 1000) when testing for association with a continuous trait for 20 individuals and 100 individuals, respectively

As expected, increasing the number of independent experimental units (e.g., individuals) in a study is the best way to increase power to detect true differences between traits measured at the individual and not individual cell level (Fig. 3a). Power calculations for binary phenotypes consisting of 10 individuals per treatment group reveal that there are only marginal gains in power when more than 100 cells per individual are sampled for a particular analysis unit (Fig. 3b). We also note that methods that do not account for within person correlation grossly overestimate power. For example, when estimating power with an approach that estimates the power for cells as independent units (assuming a type 1 error rate of α = 0.05, a fold change of 1.3, 10 individuals per treatment and 100 cells per individual), the power is overestimated as 0.93 instead of 0.71 when appropriately accounting for the within person correlation. Power calculations for continuous phenotypes, with the same sample sizes and constant within-person correlations among cells, demonstrate even smaller gains in power when more than 100 cells per individual are sampled for a particular analysis unit (Fig. 3c). The gains in power from sampling more cells per individual will decrease as the numbers of independent experimental units increase (Fig. 3d). This is true for both types of analyses. As the degree of correlation among cells within a person decreases and approaches zero, rarely observed, the value tradeoff between independent experimental units and individual cells will vary. Further, we note that if the cell-type of interest has much more or much less zero-inflation (i.e., less information), then the gains in power from sampling more cells may be greater or smaller, respectively. This is why estimating the data structure of the cell types of interest from preliminary data is a critically important feature of our *Hierarchicell* package. To consistently identify fold-change differences of at least 1.2 as statistically significant (power > 0.80), we approximate that researchers will need a minimum of 40 samples per group and 100 cells per sample in a classical case/control design. To consistently identify genes correlated with a correlation coefficient of 0.4 with a phenotype (power > 0.80), we approximate that researchers will need a minimum of 100 samples and 100 cells per sample.

As experiments get larger, computational time will increase. Future work will parallelize the code. To more rapidly close in on plausible sample size options, a researcher can apply the aggregate (“pseudo-bulk) methods power estimates and as one approaches feasible design shift to refining the estimates using the two-part hurdle mixed model employed here. However, it is important to do this refining step given the differences between these two approaches and the types of scenarios where aggregation methods will be underpowered [7, 18].

Future iterations of this package will incorporate any novel single-cell RNA-seq differential expression methods that properly account for the within sample correlation. In addition, we will parallelize the code and improve the speed of software by building components of the software in other languages (such as C++ via rcpp), and/or storing results of a large number of scenarios for quick and easy access to the necessary information. Future developments would be to incorporate the relationship between power and the variance explained by an effect, not simply fold-change between treatments. In real data, the variances explained by an effect fluctuate greatly among genes and cell types. While the simulated expression data herein have variances that are modeled after real data and are allowed to fluctuate by genes, simulating a direct relationship between the variance and an effect will be a meaningful addition to this work.

## Conclusions

To date, none of the primary power calculation methods are directly applicable for differential expression analysis with single-cell RNA-seq data. Here, we present an R-package, *Hierarchicell*, with two purposes: 1) simulation of hierarchical single-cell RNA-seq data, and 2) computation of power estimates using a mixed-effects models for testing hypotheses of differential gene expression in single-cell RNA-seq data. *Hierarchicell* allows for a range of inputs and parameter settings and even the evaluation of various single-cell specific methods, but encourages using linear mixed models with individual as a random effect for both binary and continuous outcomes, as implemented in MAST [7, 19]. We recommend these mixed effects models because they retain appropriate type 1 error rates while maintaining power. Proper calculation of statistical power coupled with proper analysis methods that account for the correlation among cells from the same individual will increase robustness and reproducibility of single-cell studies, thereby reducing the cost while accelerating the rate of scientific discovery.

## Availability and requirements

- Project name: hierarchicell
- Project home page: https://github.com/kdzimm/hierarchicell
- Operating system(s): Linux, Mac, and PC
- Programming language: R
- Other requirements: No
- License: CC0
- Any restrictions to use by non-academics: No

## Supplementary Information

**Additional file 1.**

## Data Availability

The data used for the simulated data herein and in the long-form documentation (R vignette) are available under the accession number E-MTAB-5061 at https://www.ebi.ac.uk/arrayexpress/experiments/E-MTAB-5061/. The R-package is freely available on GitHub at https://github.com/kdzimm/hierarchicell (DOI: 10.5281/zenodo.4608738).

## Abbreviations

RNA-Seq: Sequencing technique which uses next-generation sequencing to reveal the presence and quantity of RNA
MAST: Model-based Analysis of Single-cell Transcriptomics
