# Supplementary material for: Hierarchicell: an R-package for estimating power for tests of differential expression with single-cell data
Source: BMC Genomics. 2021 May 1;22:319. doi: 10.1186/s12864-021-07635-w (PMC8088563; doi:10.1186/s12864-021-07635-w)
Supplement: Supplementary file 1 — Additional file 1. [file 12864_2021_7635_MOESM1_ESM.pdf]

Hierarchicell Vignette

Kip D Zimmerman and Carl D. Langefeld

2021-03-24

Overview

This vignette is designed to teach users how to use the hierarchicell package to take a real preliminary single-cell RNA-seq dataset, simulate data that recapitulates the most important characteristics of that real data, and use that simulated data to estimate the power with a two-part hurdle mixed model.

The importance of hierarchicell

Single-cell data has a hierarchical structure that is both intuitive and can also be seen empirically. Because of the shared genetic and environmental background that is shared among cells sampled from the same individual, it can be demonstrated that cells (of the same cell type) sampled from the same individual are more correlated than cells sampled from different individuals (Zimmerman et al., 2021). This is important because it means that single-cell data have a hierarchical structure that is rarely accounted for in the statistical analysis of single-cell data and in the study design of single-cell experiments. The manuscript titled “A practical solution to pseudoreplication bias in single-cell studies” (Zimmerman et al., 2021) demonstrates why failing to account for the intra-individual correlation structure in single-cell RNA-seq studies leads to severely inflated type 1 error rates. However, the majority of single-cell’s differential expression methods treat cells as if they were independent. Recently, more researchers have implemented methods that account for the correlation structure and have properly identified the true independent experimental unit (i.e., the individual rather than the cells) in their analyses. However, a vast majority of studies are still treating cells independently or have been designed in a manner (e.g., one independent sample per treatment group with 1000s of cells) that make proper statistical inference impossible.

Other power calculators and simulation engines that currently exist simulate cells independently. They provide guidance about the number of cells that required to meet a certain power threshold. Here, we provide guidance about the number of independent individuals (as well as the number of cells nested within those individuals) that are needed to reach a certain power threshold. This distinction is extremely important - thoughtfully assessing how many independent experimental samples (i.e., individuals) are needed for a study is critical to effective study design.

Why mixed models?

When a dataset contains multiple levels (i.e., a hierarchy) the variability can be split into between group variability and within group variability. With such data, units at the highest level (i.e., individuals) are the only truly independent units and treating any of the units of subsequent levels (i.e., cells) as independent is statistically inappropriate. There is a long body of literature that demonstrates that applying statistical inference to replicates that are not statistically independent without properly accounting for their correlation structure will inflate type 1 error rates and lead to spurious results. As the denominator of most statistical tests is a function of the variance, not accounting for the positive correlation among sampling units underestimates the true standard error and leads to false positives. In addition, treating each replicate as

independent inflates the test degrees of freedom, making it easier to falsely reject the null hypothesis.

Here, we implement mixed models as a means of handling the correlation structure in single-cell RNA-seq data. A different, but commonly applied method that is a valid approach for handling the heirarchical nature of single-cell data, is aggregating the gene expression values from each of the cells within an individual and then computing the analysis on the aggregate values. Doing so, however, reduces the number of data points and is a loss of information which makes this a conservative approach. Overall, it has been demonstrated repeatedly in the literature that mixed models lead to the most accurate results when analyzing hierarchical data.

Mixed models are implemented as a means of accounting for correlated data by modeling the hierarchical structure of the data by partitioning out the between and within group variability. Mixed models are an extension of typical linear models that allow for both fixed and random effects. A fixed effect is a parameter that does not vary, but random effects are parameters that are themselves random variables. A fixed effect model (think typical linear regression) assumes the data are random variables but the parameters are fixed. With a random effect, however, the data are random variables and the parameters are random variables at the lower level, but fixed at the highest level. For example, if we were to model the test scores of students from six different schools based on some predictor variable, the data would have two levels: schools and students within those schools. The overall mean test scores across all students and all schools is fixed, but the parameter within each school is assumed to follow a random normal distribution with the same mean as the overall mean and some variance.

Why the two part hurdle model?

The two-part hurdle model is implemented in a tool called MAST (Finak et al., 2015) and it is used to simultaneously model the continuous (non-zero values) and discrete ("expressed"/"not expressed") components of single-cell data. This is an excellent model for identifying genes that are either:

1. expressed at varying magnitudes (e.g., average expression of 100 vs average expression of 1000)
2. expressed at different rates (e.g., 30% of cells expressing the gene being tested vs 60% of cells expressing the same gene)
3. some combination of both

Other models are primarily testing the first hypothesis, but we believe the second hypothesis is just as meaningful and will allow users to capture more genes.

Installation and R setup

Before beginning, you will need to install the hierarchicell package This can be done by:

- install.packages() from CRAN
- devtools::install_github() from GitHub (this will download the package in development, so will offer latest developments, but may be unstable)

To load the package simply type "library(hierarchicell)". This will attach the package and all of its related functions.It is important to install and load all of hierarchicell's dependencies as well.

```
## To install from CRAN
#install.packages("hierarchicell")

## To install from github
```

```r
#devtools::install_github("kdzimm/hierarchicell")

## Load dependencies (install where necessary)

#suppressWarnings(suppressPackageStartupMessages({
#    library(fitdistrplus)
#    library(ggplot2)
#    library(MASS)
#    library(tidyr)
#    library(gdata)
#    library(Seurat)
#    library(data.table)
#    library(EnvStats)
#    library(purrr)
#    library(dplyr)
#    library(MAST)
#    library(SummarizedExperiment)
#    library(BiocGenerics)
#    library(ROTS)
#    library(VGAM)
#    library(geepack)
#    library(glmmTMB)
#    library(sva)
#    library(monocle)
#    library(stats)
#    library(methods)
#    library(grDevices)
#    library(DESeq2)
#}))

## Load R package
library(hierarchicell)
```

Load and filter input data

After installing and loading the R-package, the initial steps will be to read in your data and format it properly. Then you have the option of filtering your data. By default, the program will only filter out cells and genes that contain all zero values. This is the default because, we believe that the proportion of zeros in your preliminary dataset is informative for downstream power calculations. If you expect your next set of data to contain fewer zeros or to be of higher quality, then filtering at this step may be recommended.

Data should be only for cells of the specific cell-type you are interested in simulating or computing power for. The input data will need to be a data.frame where the unique cell identifier is in column one and the sample identifier is in column two with the remaining columns all being genes. Data should be only for cells of the specific cell-type you are interested in simulating or computing power for. Data should also contain as many unique sample identifiers as possible. If you are inputing data that has less than 5 unique values for sample identifier (i.e., independent experimental units), then the empirical estimation of the inter-individual heterogeneity is going to be very unstable. Finding such a dataset will be difficult at this time, but, over time (as experiments grow in sample size and the numbers of publicly available single-cell RNAseq

datasets increase), this should improve dramatically.

If you do not have a preliminary dataset you would like to use, running the filter_counts function without specifying any options ("filter_counts()") will load a default dataset of pancreatic alpha cells. This a reasonable starting place for researchers looking to simply get an idea of power for their study.

```
## Run hierarchicell's filter_counts function to filter and prepare for next steps

gene_counts_filtered <- hierarchicell::filter_counts()
#> Filtering default dataset
#> Genes and cells have been filtered, ready for estimating parameters
gene_counts_filtered[1:5,1:5]
#>        CellID IndividualID Gene1 Gene2 Gene3
#> 2  ERR1630015           H1     0     0     0
#> 6  ERR1630021           H1     0     0     0
#> 13 ERR1630028           H1     0     0     0
#> 14 ERR1630029           H1     0     0     0
#> 15 ERR1630030           H1     0     0     0
```

Estimate and plot various parameters

After running the filter_counts function and ensuring your data is in the proper format, the next step involves estimation of the simulation parameters. The functions estimate the empirical distributions for dropout rate, global gene means, and they model the hierarchical variance structure of the input data. The default for these functions assumes the data are normalized somehow (i.e., "TPM", "RPM","FPKM"), however, raw data can be input as well. It will just need to be specified (type = "Raw").

Here, we will run the global compute_data_summaries function first. This is the only function you will need to run to continue on with next steps. In the latter steps we will use the individual functions within the compute_data_summaries function to plot distributions and visualize how well the program is modeling the behavior of the input data. This is recommended for you to ensure nothing extremely concerning is happening with your data.

NOTE: With some data types, the intra-individual variance is (more often than not) smaller than the intra-individual mean. This leads to negative dispersion estimates which end up being disregarded. We are working on developing more advanced simulation methods that take this into account and use a poisson or gaussian instead of a negative binomial where these scenarios exist.

```
## Compute data summaries - all-in-one step

data_summ <- hierarchicell::compute_data_summaries(gene_counts_filtered, type = "Norm")
#> Computing sample means, dropout rates, and dispersion ...
#> Computing final data summaries ...

## Example with "raw" counts
# data_summ <- hierarchicell::compute_data_summaries(gene_counts_filtered, type = "Raw")

## Examine how well the program is modeling the behavior of the input data
```

```
## Histogram of the gene means

approximate_gene_mean(data_summ, plot = TRUE)
#> Plotting distribution of grand means
#> Warning: Ignoring unknown parameters: binwidth, bins, pad
```

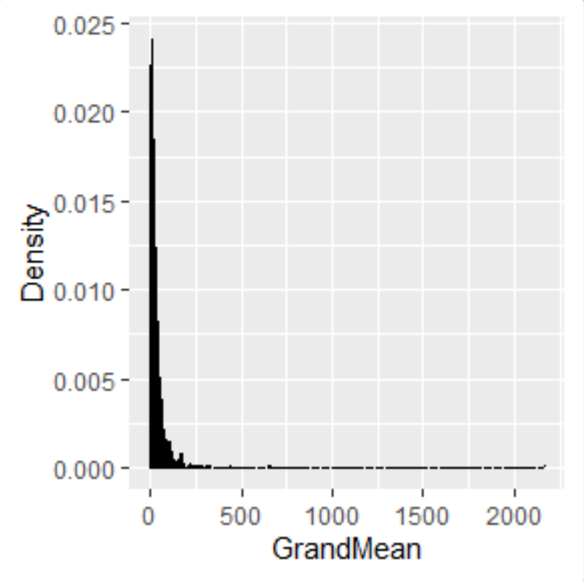


```
#> [1] 0.70531567 0.01832447

## Model dispersion as a function of the grand mean

model_dispersion(data_summ, plot = TRUE)
#> Plotting dispersion
```

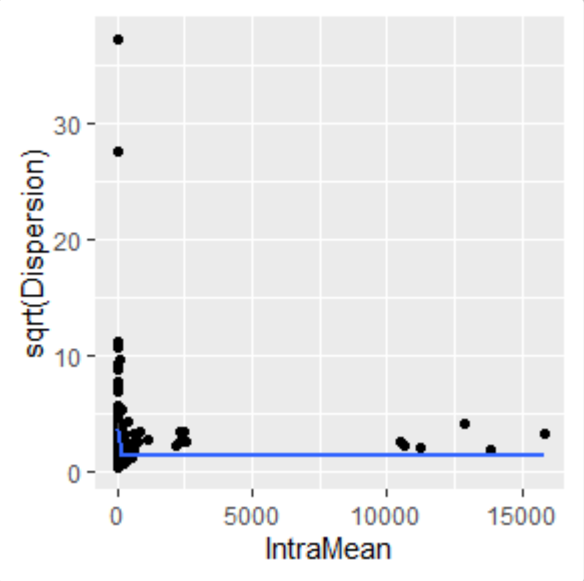


```
#> [1] 1.054395 1.610248

## Model inter-individual variance as a function of the grand mean
```

```
model_inter(data_summ, plot = TRUE)
#> Plotting inter-individual standard deviation
```

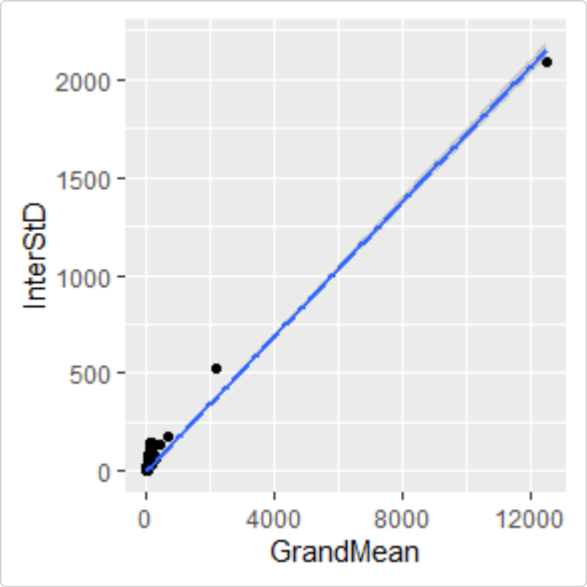


```
#> [1] 0.1727052

## Histogram of mean dropout

approximate_gene_drop(data_summ, plot = TRUE)
#> Plotting distribution of dropout
#> Warning: Ignoring unknown parameters: binwidth, bins, pad
```

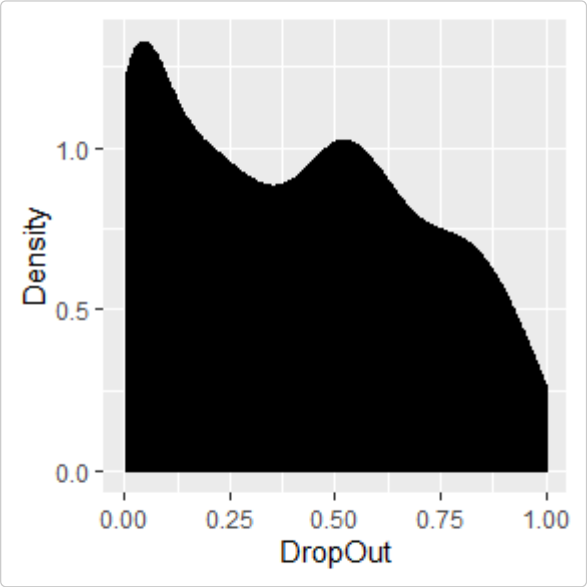

```
#> [1] 0.7502302 1.9042279

## Model dropout variance as a function of mean dropout

model_drop_sd(data_summ, plot = TRUE)
#> Plotting dropout
```

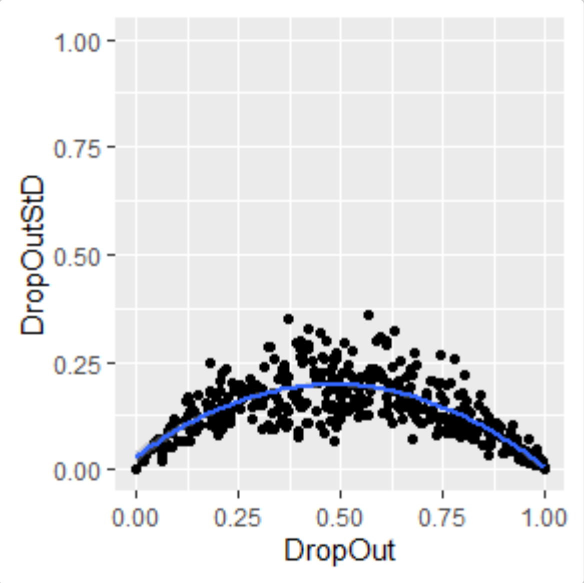


```
#> [1]  0.03029633  0.69215768 -0.71778302
```

Simulate data and visualize

The following function is only used if you are interested in simulating data, without using the data to estimate type 1 error or power. It is also useful if you want to visualize your simulated data with a tSNE plot. The main component to check for is a hierarchical structure of some degree (i.e.,cells from the same individual are clustering together more closely to one another). Depending on the cell type, however, the degree of intra-individual correlation will vary greatly, however, so an initial tSNE plot of the real data might be worth observing as well.

```
## Simulate your data and store it

sim_dat1 <- simulated_macroph <- simulate_hierarchicell(data_summ,
                                           n_genes = 100, #100 genes
                                           n_per_group = 5, #5 individuals per group
                                           cells_per_control = 50, #50 cells per control
                                           cells_per_case = 50, #50 cells per case
                                           ncells_variation_type = "Poisson", #Cells per
        individual
                                           foldchange = 2) #Fold change
#> Computing simulation parameters ...
#> ------------------------------------------------------
#> Distribution of grand means is a gamma
```

```
#> with shape: 0.71 and rate: 0.02
#> -------------------------------------------------------
#> Distribution for gene-wise dropout is a gamma
#>  with shape: 0.75 and rate: 1.9
#> -------------------------------------------------------
#> Function for dropout SD is:
#> DropoutStD = 0.03 + 0.69*DropOut + -0.72*(DropOut**2)
#> -------------------------------------------------------
#> Function for inter-individual SD is:
#> InterStDev = 0 + 0.17*GrandMean)
#> -------------------------------------------------------
#> Function for dispersion is:
#>  exp(1.05 + 1.61/IntraMean)
#> -------------------------------------------------------
#> Simulating cells ...
#> -------------------------------------------------------
#> Simulating expression values ...
#> -------------------------------------------------------
#> All done!

## Simulate your data and visualize with a tSNE plot

sim_dat2 <- simulate_hierarchicell(data_summ,
                       n_genes = 1000,
                       n_per_group = 5,
                       cells_per_control = 50,
                       cells_per_case = 50,
                       ncells_variation_type = "Poisson",
                       foldchange = 2,
                       tSNE_plot = TRUE)
#> Computing simulation parameters ...
#> -------------------------------------------------------
#> Distribution of grand means is a gamma
#> with shape: 0.71 and rate: 0.02
#> -------------------------------------------------------
#> Distribution for gene-wise dropout is a gamma
#>  with shape: 0.75 and rate: 1.9
#> -------------------------------------------------------
#> Function for dropout SD is:
#> DropoutStD = 0.03 + 0.69*DropOut + -0.72*(DropOut**2)
#> -------------------------------------------------------
#> Function for inter-individual SD is:
#> InterStDev = 0 + 0.17*GrandMean)
#> -------------------------------------------------------
#> Function for dispersion is:
#>  exp(1.05 + 1.61/IntraMean)
#> -------------------------------------------------------
#> Simulating cells ...
#> -------------------------------------------------------
#> Simulating expression values ...
```

```
#> ----------------------------------------------------------
#> Generating tSNE plot ...
#> Warning: The following arguments are not used: do.plot
#> Centering and scaling data matrix
#> PC_ 1
#> Positive:  Gene290, Gene179, Gene884, Gene922, Gene259, Gene103, Gene405, Gene979, Gene556,
      Gene406
#>     Gene752, Gene126, Gene856, Gene778, Gene925, Gene644, Gene157, Gene846, Gene65, Gene896
#>     Gene167, Gene728, Gene107, Gene994, Gene722, Gene875, Gene768, Gene641, Gene897, Gene377
#> Negative:  Gene777, Gene238, Gene145, Gene708, Gene77, Gene156, Gene883, Gene869, Gene675,
      Gene530
#>     Gene628, Gene623, Gene49, Gene123, Gene692, Gene539, Gene714, Gene585, Gene284, Gene235
#>     Gene524, Gene676, Gene898, Gene296, Gene823, Gene90, Gene291, Gene834, Gene519, Gene433
#> PC_ 2
#> Positive:  Gene473, Gene985, Gene906, Gene457, Gene170, Gene423, Gene435, Gene816, Gene249,
      Gene629
#>     Gene11, Gene717, Gene690, Gene221, Gene208, Gene201, Gene988, Gene378, Gene410, Gene912
#>     Gene166, Gene999, Gene310, Gene768, Gene376, Gene831, Gene696, Gene426, Gene536, Gene783
#> Negative:  Gene71, Gene950, Gene42, Gene661, Gene171, Gene605, Gene237, Gene977, Gene886,
      Gene295
#>     Gene76, Gene92, Gene83, Gene548, Gene390, Gene860, Gene628, Gene302, Gene213, Gene433
#>     Gene967, Gene416, Gene694, Gene670, Gene750, Gene538, Gene236, Gene202, Gene145, Gene916
#> PC_ 3
#> Positive:  Gene797, Gene260, Gene748, Gene200, Gene965, Gene129, Gene516, Gene312, Gene705,
      Gene955
#>     Gene132, Gene394, Gene855, Gene644, Gene285, Gene662, Gene559, Gene973, Gene100, Gene500
#>     Gene821, Gene441, Gene249, Gene242, Gene953, Gene513, Gene412, Gene118, Gene820, Gene926
#> Negative:  Gene109, Gene524, Gene272, Gene138, Gene521, Gene829, Gene996, Gene828, Gene298,
      Gene371
#>     Gene747, Gene90, Gene788, Gene930, Gene988, Gene717, Gene570, Gene526, Gene628, Gene637
#>     Gene320, Gene438, Gene173, Gene664, Gene698, Gene887, Gene43, Gene963, Gene878, Gene784
#> PC_ 4
#> Positive:  Gene798, Gene511, Gene656, Gene113, Gene813, Gene468, Gene101, Gene386, Gene404,
      Gene283
#>     Gene906, Gene351, Gene56, Gene510, Gene359, Gene413, Gene176, Gene474, Gene465, Gene845
#>     Gene61, Gene39, Gene318, Gene878, Gene407, Gene914, Gene859, Gene553, Gene310, Gene560
#> Negative:  Gene611, Gene774, Gene912, Gene311, Gene614, Gene797, Gene341, Gene391, Gene760,
      Gene154
#>     Gene66, Gene428, Gene632, Gene682, Gene901, Gene476, Gene358, Gene713, Gene423, Gene8
#>     Gene541, Gene825, Gene620, Gene68, Gene995, Gene266, Gene558, Gene622, Gene35, Gene932
#> PC_ 5
#> Positive:  Gene778, Gene70, Gene552, Gene237, Gene559, Gene719, Gene512, Gene460, Gene947,
      Gene599
#>     Gene974, Gene743, Gene488, Gene827, Gene878, Gene146, Gene263, Gene927, Gene448, Gene127
#>     Gene606, Gene531, Gene159, Gene333, Gene264, Gene767, Gene910, Gene852, Gene687, Gene818
#> Negative:  Gene408, Gene467, Gene371, Gene374, Gene476, Gene773, Gene695, Gene673, Gene13,
      Gene136
#>     Gene231, Gene164, Gene154, Gene706, Gene252, Gene608, Gene934, Gene468, Gene566, Gene72
#>     Gene85, Gene137, Gene980, Gene595, Gene120, Gene287, Gene428, Gene766, Gene548, Gene411
#> Computing nearest neighbor graph
#> Computing SNN
#> Modularity Optimizer version 1.3.0 by Ludo Waltman and Nees Jan van Eck
#>
```

```
#> Number of nodes: 516
#> Number of edges: 24719
#>
#> Running Louvain algorithm...
#> Maximum modularity in 10 random starts: 0.5991
#> Number of communities: 2
#> Elapsed time: 0 seconds
#> ------------------------------------------------------
#> All done!
```

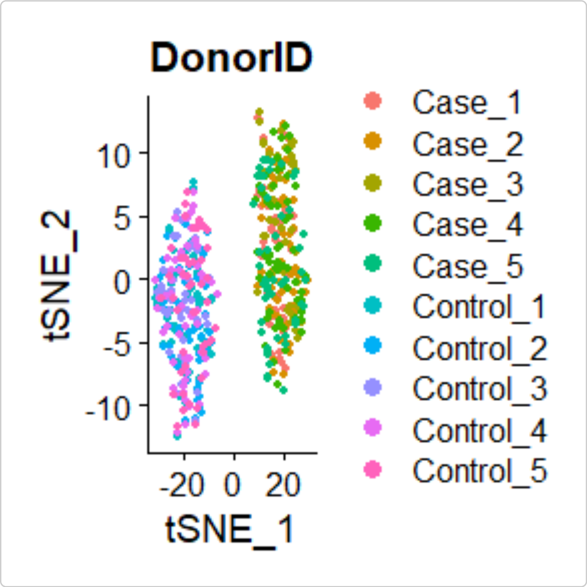


```
## Simulate continuous outcomes and visualize with a tSNE plot

sim_dat3 <- simulate_hierarchicell_continuous(data_summ,
                                  n_genes = 1000,
                                  n_individuals = 10,
                                  cells_per_individual = 50,
                                  ncells_variation_type = "Poisson",
                                  rho = 1,
                                  continuous_mean = 0,
                                  continuous_sd = 1,
                                  decrease_dropout = 0,
                                  tSNE_plot = TRUE)
#> Computing simulation parameters ...
#> ------------------------------------------------------
#> Distribution of grand means is a gamma
#> with shape: 0.71 and rate: 0.02
#> ------------------------------------------------------
#> Distribution for gene-wise dropout is a gamma
#>  with shape: 0.75 and rate: 1.9
#> ------------------------------------------------------
#> Function for dropout SD is:
#> DropoutStD = 0.03 + 0.69*DropOut + -0.72*(DropOut**2)
```

```
#> -------------------------------------------------------
#> Function for inter-individual SD is:
#> InterStDev = 0 + 0.17*GrandMean)
#> -------------------------------------------------------
#> Function for dispersion is:
#>  exp(1.05 + 1.61/IntraMean)
#> -------------------------------------------------------
#> Simulating cells ...
#> -------------------------------------------------------
#> Simulating expression values ...
#> -------------------------------------------------------
#> Generating tSNE plot ...
#> Warning: The following arguments are not used: do.plot
#> Centering and scaling data matrix
#> PC_ 1
#> Positive:  Gene564, Gene471, Gene820, Gene864, Gene955, Gene615, Gene116, Gene214, Gene98,
       Gene89
#>     Gene14, Gene972, Gene994, Gene687, Gene445, Gene673, Gene934, Gene948, Gene674, Gene117
#>     Gene909, Gene93, Gene492, Gene675, Gene877, Gene232, Gene141, Gene676, Gene858, Gene105
#> Negative:  Gene270, Gene67, Gene257, Gene212, Gene641, Gene327, Gene519, Gene320, Gene469,
       Gene319
#>     Gene648, Gene473, Gene207, Gene561, Gene313, Gene988, Gene431, Gene430, Gene768, Gene991
#>     Gene63, Gene119, Gene874, Gene91, Gene533, Gene797, Gene343, Gene719, Gene346, Gene616
#> PC_ 2
#> Positive:  Gene424, Gene821, Gene477, Gene802, Gene491, Gene335, Gene374, Gene474, Gene888,
       Gene631
#>     Gene698, Gene459, Gene921, Gene161, Gene289, Gene596, Gene940, Gene385, Gene981, Gene591
#>     Gene239, Gene600, Gene593, Gene782, Gene399, Gene29, Gene299, Gene521, Gene260, Gene644
#> Negative:  Gene930, Gene664, Gene661, Gene768, Gene791, Gene397, Gene37, Gene780, Gene2,
       Gene503
#>     Gene875, Gene96, Gene157, Gene836, Gene795, Gene223, Gene967, Gene36, Gene858, Gene203
#>     Gene90, Gene706, Gene983, Gene160, Gene853, Gene268, Gene869, Gene35, Gene562, Gene445
#> PC_ 3
#> Positive:  Gene991, Gene310, Gene144, Gene578, Gene366, Gene786, Gene802, Gene971, Gene90,
       Gene234
#>     Gene860, Gene158, Gene327, Gene424, Gene817, Gene405, Gene979, Gene613, Gene902, Gene36
#>     Gene191, Gene290, Gene203, Gene251, Gene956, Gene118, Gene45, Gene416, Gene496, Gene983
#> Negative:  Gene554, Gene623, Gene143, Gene484, Gene923, Gene4, Gene80, Gene92, Gene238, Gene758
#>     Gene598, Gene325, Gene712, Gene944, Gene782, Gene263, Gene643, Gene432, Gene677, Gene139
#>     Gene546, Gene209, Gene9, Gene731, Gene716, Gene866, Gene651, Gene616, Gene820, Gene426
#> PC_ 4
#> Positive:  Gene576, Gene24, Gene756, Gene898, Gene157, Gene858, Gene742, Gene90, Gene316,
       Gene195
#>     Gene868, Gene94, Gene554, Gene589, Gene928, Gene877, Gene957, Gene364, Gene441, Gene853
#>     Gene893, Gene155, Gene697, Gene527, Gene98, Gene643, Gene936, Gene764, Gene58, Gene879
#> Negative:  Gene415, Gene125, Gene167, Gene653, Gene703, Gene170, Gene814, Gene615, Gene783,
       Gene102
#>     Gene294, Gene841, Gene980, Gene92, Gene574, Gene591, Gene832, Gene477, Gene307, Gene826
#>     Gene587, Gene183, Gene242, Gene731, Gene440, Gene258, Gene116, Gene253, Gene974, Gene706
#> PC_ 5
#> Positive:  Gene159, Gene37, Gene476, Gene552, Gene436, Gene863, Gene816, Gene812, Gene340,
       Gene679
#>     Gene623, Gene46, Gene994, Gene192, Gene345, Gene369, Gene250, Gene418, Gene132, Gene998
```

```
#>     Gene845, Gene448, Gene820, Gene925, Gene98, Gene95, Gene477, Gene113, Gene723, Gene485
#> Negative:  Gene387, Gene557, Gene41, Gene451, Gene99, Gene221, Gene641, Gene441, Gene885,
        Gene388
#>     Gene352, Gene305, Gene231, Gene644, Gene145, Gene859, Gene236, Gene774, Gene426, Gene161
#>     Gene528, Gene322, Gene750, Gene341, Gene469, Gene111, Gene983, Gene408, Gene337, Gene254
#> Computing nearest neighbor graph
#> Computing SNN
#> Modularity Optimizer version 1.3.0 by Ludo Waltman and Nees Jan van Eck
#>
#> Number of nodes: 487
#> Number of edges: 22052
#>
#> Running Louvain algorithm...
#> Maximum modularity in 10 random starts: 0.3740
#> Number of communities: 5
#> Elapsed time: 0 seconds
#> ------------------------------------------------------
#> All done!
```

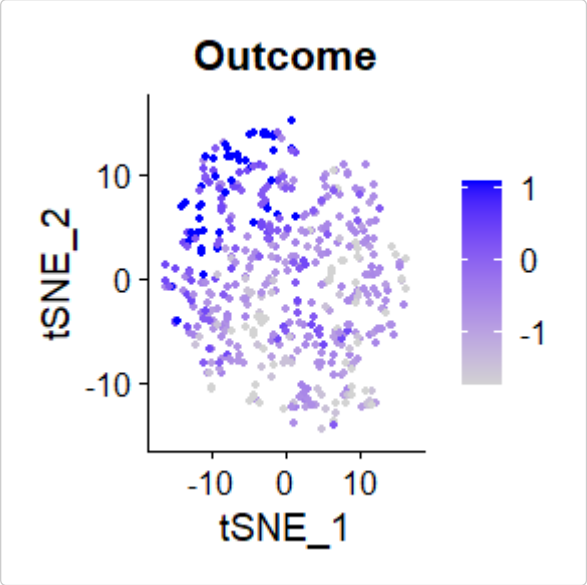

Estimate power for various scenarios

Once the other necessary steps have been completed (minimally, 1. filter_counts 2. compute_data_summaries), the power_hierarchicell function can be used to estimate power for a variety of scenarios. This function can take some time, because it is analyzing each gene in the simulated data with linear mixed effects model. To save time, one can apply the pseudobulk methods from the type 1 error calculator (error_hierarchicell(method = “Pseudobulk_mean”)) and still obtain a reasonable approximation of power for a given fold change. However, if the numbers of cells per individual (ncells_variation_type) are modeled with a negative binomial where there is extreme imbalance this method will be underpowered.

```
## Estimate power for 10 individuals per treatment group with a fold-change of 2 (aggregation
        methods)
## NOTE: This will throw an error for using a fold-change not equal to 1, this is fine, because
        you are not using the tool in this instance to compute type 1 error.
```

```r
if (identical(Sys.getenv("NOT_CRAN", unset = "true"), "true")) {

  error_hierarchicell(data_summ,
                     method = "Pseudobulk_mean",
                     n_genes = 1000,
                     n_per_group = 10,
                     cells_per_case = 50,
                     cells_per_control = 50,
                     ncells_variation_type = "Poisson",
                     pval = 0.05,
                     foldchange = 2)

## Estimate power for 10 individuals per treatment group with a fold-change of 2 (mixed models)

error_hierarchicell(data_summ,
                     method = "MAST_RE",
                     n_genes = 1000,
                     n_per_group = 10,
                     cells_per_case = 50,
                     cells_per_control = 50,
                     ncells_variation_type = "Poisson",
                     pval = 0.05,
                     foldchange = 2)

## Or:

power_hierarchicell(data_summ,
                     n_genes = 1000,
                     n_per_group = 10,
                     cells_per_case = 50,
                     cells_per_control = 50,
                     ncells_variation_type = "Poisson",
                     pval = 0.05,
                     foldchange = 2)

## Estimate power for 40 individuals with a continuous outcome (mean = 10, sd = 4) that is highly
         correlated to gene expression (rho = 0.99)

power_hierarchicell_continuous(data_summ,
                                n_genes = 1000,
                                n_individuals = 20,
                                cells_per_individual = 50,
                                ncells_variation_type = "Poisson",
                                rho = 0.99,
                                continuous_mean = 10,
                                continuous_sd = 4)

## Estimate power for 40 individuals with a continuous outcome that is moderately correlated to
         gene expression (rho = 0.6)
```

```r
power_hierarchicell_continuous(data_summ,
                               n_genes = 1000,
                               n_individuals = 20,
                               cells_per_individual = 50,
                               ncells_variation_type = "Poisson",
                               rho = 0.6,
                               continuous_mean = 10,
                               continuous_sd = 4)

}
#> -----------------------------------------------
#> Foldchange is not equal to 1, you are not simulating under the null.
#> For type 1 error rates, please keep foldchange equal to 1
#> -----------------------------------------------
#> Type 1 error for 0.05 is: 0.961187214611872
#> -----------------------------------------------
#> Foldchange is not equal to 1, you are not simulating under the null.
#> For type 1 error rates, please keep foldchange equal to 1
#> -----------------------------------------------
#> Warning in vcov.merMod(object@fitD): variance-covariance matrix computed from finite-difference
      Hessian is
#> not positive definite or contains NA values: falling back to var-cov estimated from RX
#> Warning in checkConv(attr(opt, "derivs"), opt$par, ctrl = control$checkConv, :
#> Model failed to converge with max|grad| = 0.0155403 (tol = 0.002, component 1)
#> Warning in checkConv(attr(opt, "derivs"), opt$par, ctrl = control$checkConv, :
#> Model failed to converge with max|grad| = 0.0210279 (tol = 0.002, component 1)
#> Type 1 error for 0.05 is: 0.980952380952381
#>
#> Done!
#> Combining coefficients and standard errors
#> Calculating log-fold changes
#> Calculating likelihood ratio tests
#> Refitting on reduced model...
#>
#> Done!
#> Continuous Power for 0.05 is: 0.997530864197531
#> Hurdle Power for 0.05 is: 0.992592592592593
#> Discrete Power for 0.05 is: 0.0748792270531401
#>
#> Done!
#> Combining coefficients and standard errors
#> Calculating log-fold changes
#> Calculating likelihood ratio tests
#> Refitting on reduced model...
#>
#> Done!
#> Continuous Power for 0.05 is: 1
#> Hurdle Power for 0.05 is: 1
#> Discrete Power for 0.05 is: 0.0796252927400468
#>
#> Done!
```

```
#> Combining coefficients and standard errors
#> Calculating log-fold changes
#> Calculating likelihood ratio tests
#> Refitting on reduced model...
#>
#> Done!
#> Continuous Power for 0.05 is: 0.956120092378753
#> Hurdle Power for 0.05 is: 0.829099307159353
#> Discrete Power for 0.05 is: 0.0666666666666667
```
